# Primary Cervical Leiomyoma with Remarkable Calcification and Ossification

**DOI:** 10.1155/2014/896275

**Published:** 2014-02-18

**Authors:** Takahiro Yamanishi, Kaname Sakamoto, Hiroyuki Watanabe, Takaaki Yonaga, Naoki Oishi, Ryohei Katoh, Keisuke Masuyama

**Affiliations:** ^1^Department of Otolaryngology-Head and Neck Surgery, Faculty of Medicine, University of Yamanashi, 1110 Shimokato, Chuo, Yamanashi 409-3898, Japan; ^2^Department of Pathology, Faculty of Medicine, University of Yamanashi, 1110 Shimokato, Chuo, Yamanashi 409-3898, Japan

## Abstract

We encountered a patient with primary cervical leiomyoma with remarkable calcification and ossification. A 68-year-old man presenting with induration and swelling of the left submandibular region was found to have nodular lesions with calcifications in the left submandibular region and the upper mediastinum on CT. Fine needle aspiration biopsies (FNAB) of the left submandibular lesion revealed no malignancy. Resection was performed for definitive diagnosis and treatment. The resected specimen contained a solid tumor, which was markedly calcified and ossified on the cut surface. Histopathological examination showed proliferating spindle cells in a tangled and crossed arrangement. Immunohistochemically, the spindle cells were stained intensely with *α*-SMA and h-caldesmon, consistent with smooth muscle cells. These findings led to a definitive diagnosis of leiomyoma with calcification and ossification. This is extremely rare and the preoperative differentiation from other tumors of the head and neck was very difficult. By resection of the submandibular tumor, both definitive diagnosis of leiomyoma by histopathological and immunohistochemical analyses and treatment could be carried out. However, as the tumor in the upper mediastinum was most likely to be leiomyoma with calcification, he did not wish to undergo its biopsy and resection immediately. We have continued the follow-up.

## 1. Introduction

Leiomyoma is a benign and nonepithelial tumor that commonly arises from the uterus, esophagus, and skin. Primary leiomyomas of the head and neck account for 12% of all leiomyomas [1] and a very small percentage of all head and neck tumors. They are usually solitary, rounded, and well-demarcated masses, but primary cervical lesion with calcification and ossification is extremely rare. It is not clear that calcification and ossification are strongly suggestive of benignancy. In particular, the differentiation between leiomyoma and leiomyosarcoma is very important but often difficult at the preoperative stage [2–4]. Histopathological and immunohistochemical analyses after surgery are necessary for the definitive diagnosis of leiomyoma. Here, we report a rare case of primary cervical leiomyoma with remarkable calcification and ossification, with a review of the literature.

## 2. Case Report

The patient was a 68-year-old man presenting with induration and swelling of the left submandibular region. His past medical history and familial history were unremarkable. At the age of about 25 years, he had noticed a small induration with an irregular surface in this region, but it was left untreated because of a lack of subjective symptoms. However, it also showed no tendency to improve, and then the swelling gradually worsened.

**Figure 1:**
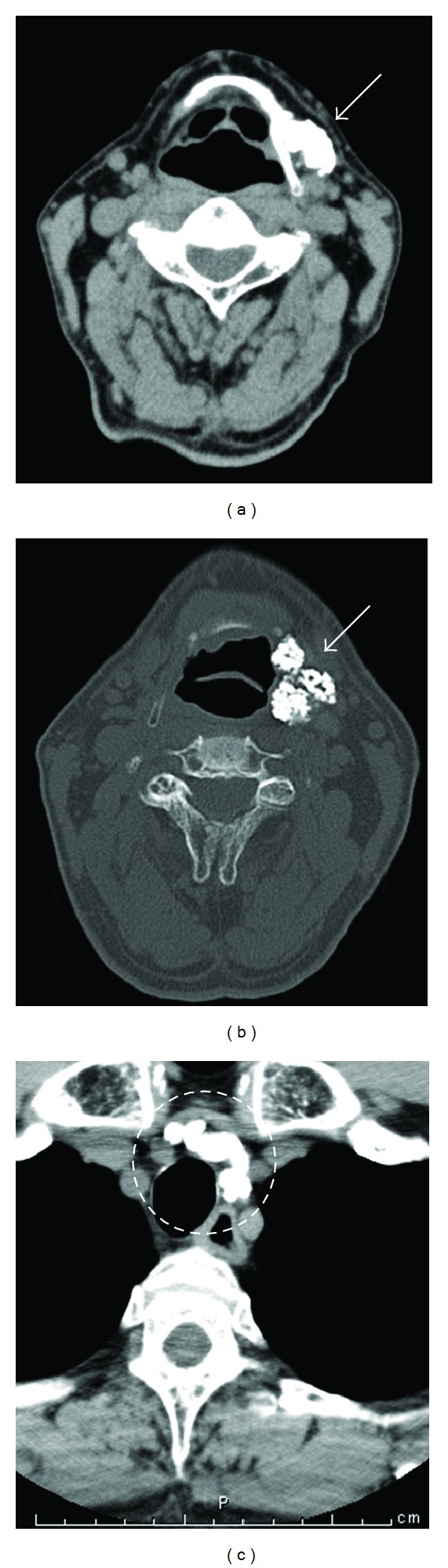
Neck and chest CT. Nodular shadows with calcification were observed (arrows in (a) and (b) and circles in (c)). ((a), (b)) Submandibular region. (c) Upper mediastinum.

At the time of his first visit to our department, except for the induration and swelling in the left submandibular region, there were no abnormal findings in the ear, nose, throat, head, and neck. CT revealed nodular shadows with marked calcifications in the left submandibular region and the upper mediastinum (Figure 1). No significant abnormalities were noted in the laboratory examinations. Preoperative FNAB of the left submandibular lesion was performed three times but revealed no evidence of malignancy.

**Figure 2:**
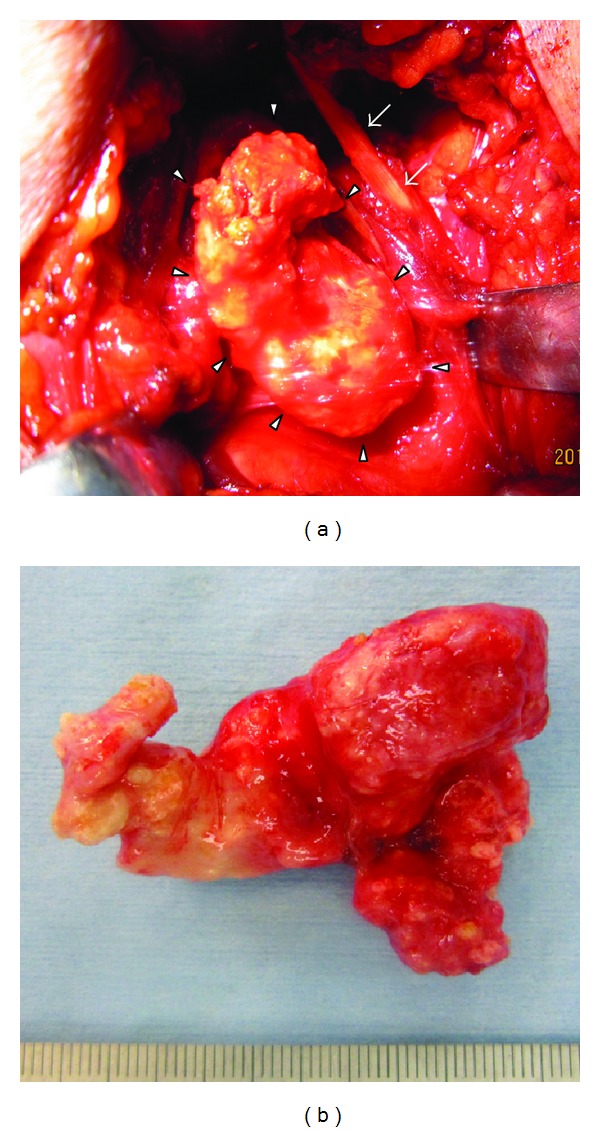
Operative findings. (a) Resection of the tumor (arrowheads). Arrows indicate the posterior belly of the digastric muscle. There was no adhesion between the tumor and surrounding tissue. (b) Resected tumor.

Because of the uncertain diagnosis, surgery was performed. After a skin incision of the left submandibular region, an irregularly surfaced firm mass in the deep submandibular space was revealed (Figure 2). First, open biopsy of a portion of the mass was carried out for intraoperative histopathological diagnosis of frozen sections. It revealed calcification with no evidence of malignancy. Following this diagnosis, tumor resection was performed. The tumor could be easily dissected from the surrounding tissue and removed, since it did not adhere to the hyoid bone, pharyngeal submucosal tissue and hypoglossal nerve. This nerve and the marginal submandibular branch of the facial nerve were identified and preserved.

**Figure 3:**
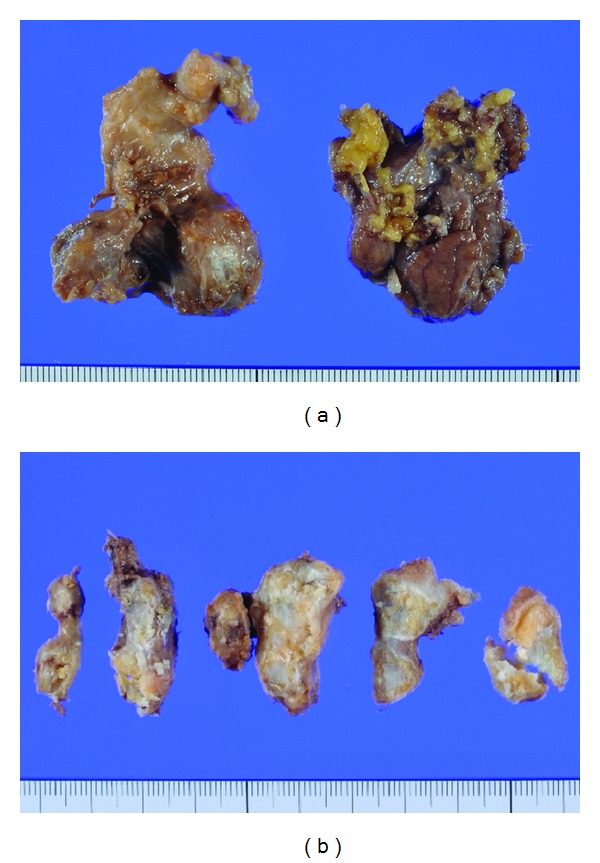
(a) Resected tumor was a 4 cm × 3 cm × 3 cm solid mass. (b) Cut surface. Lumpy calcification and ossification were noted.

**Figure 4:**
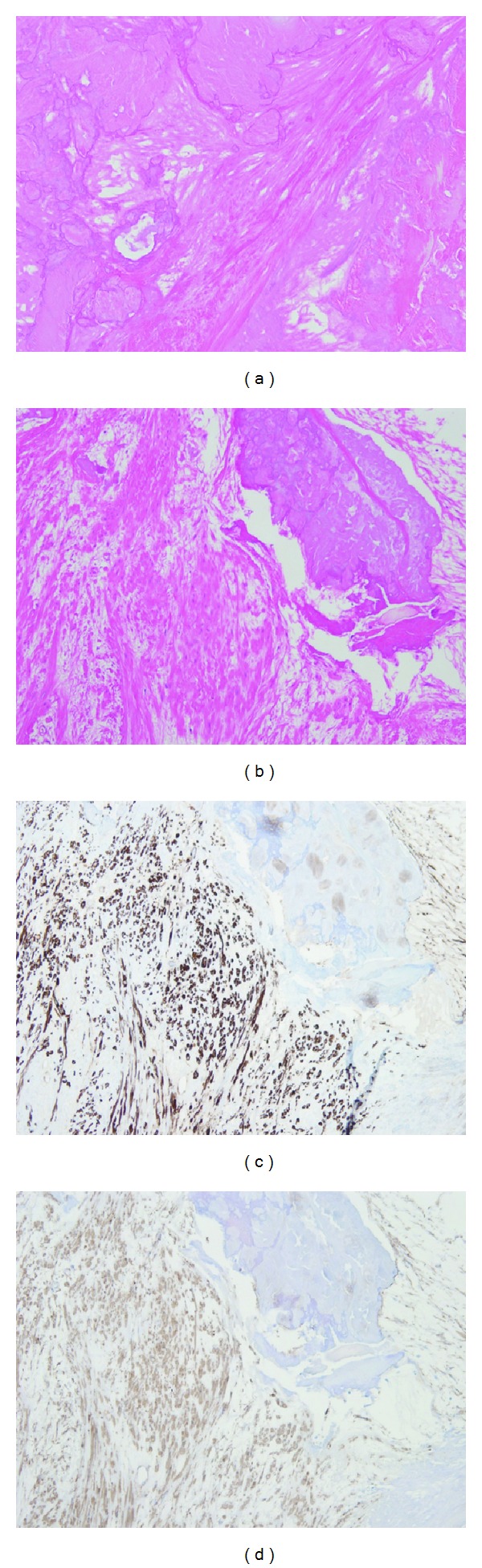
Calcification and smooth muscle cells ((a)-(b), HE stain ×200) ((c)-(d), immunohistochemical stain ((c): *α*-SMA; (d): h-caldesmon) ×200). The cells were proliferating in a tangled and crossed arrangement around calcification and stained intensely with *α*-SMA and h-caldesmon.

Grossly, the resected tumor was a 4 cm × 3 cm × 3 cm solid mass and showed marked calcification and ossification on sections (Figure 3). Histopathologically, diffuse proliferating spindle cells with eosinophilic cytoplasm were present in a tangled and crossed arrangement in and around the calcification and ossification. A histological transition was observed between the smooth muscle tissue and calcification. Immunohistochemically, the spindle cells were stained intensely with *α*-SMA and h-caldesmon, consistent with smooth muscle cells (Figure 4). These findings led to a definitive diagnosis of leiomyoma with calcification and ossification. His postoperative course was uneventful and no recurrence and no significant complications have been observed.

However, a definitive diagnosis of the mass in the upper mediastinum (Figure 1(c)) has not been obtained, it was considered most likely to be leiomyoma with calcification. Finally, the patient did not wish to undergo its resection immediately because he had no symptoms and the resection would be more invasive.

## 3. Discussion

Leiomyoma is a benign and nonepithelial tumor that commonly arises from the uterus, esophagus, and skin. Primary leiomyomas of the head and neck account for 12% of all leiomyomas [1], and paranasal sinuses are the most common primary site among them [2]. They account for a very small percentage of all head and neck tumors, and primary cervical esophageal leiomyoma constitutes only 0.3% of all esophageal leiomyomas [3]. Moreover, primary cervical leiomyoma with remarkable calcification and ossification is extremely rare, and our search of the literature revealed no such cases.

The proposed mechanisms for the development of leiomyoma include a congenital origin, blood flow disturbance, infection, and the involvement of estrogen [4], but no consensus has been reached to date. It has also been reported that progesterone receptors are expressed in the nucleus of tumor cells, and progesterone is involved in tumor development and growth [5]. This may be related to the higher incidence in females (ratio is 1 : 3.75) [6].

In the WHO classification, smooth muscle neoplasms have been classified into leiomyoma (solid leiomyoma), angiomyoma (vascular leiomyoma), and epithelioid leiomyoma (leiomyoblastoma). Leiomyoma has been shown to be the most common, and it differs from angiomyoma in terms of the degree of angiogenesis in the tissue [7]. There is support for the view that leiomyoma in the head and neck often arises from the vascular smooth muscle, the main component of the wall of small blood vessels [1, 4]. However, there is another opinion that multipotent mesenchymal cells are also its origin [8], but the histogenesis remains controversial.

The mechanisms of development of calcification with leiomyoma and multioccurrence of calcification are largely unknown. Calcification is frequently found in cells with nuclear atypia and degenerative changes [9]. The mechanisms of this include (1) secondary changes due to tissue degeneration and necrosis, (2) metabolic disorders such as parathyroid dysfunction, and (3) developmental anomalies such as teratomas [10]. There were no findings strongly suggestive of (2) or (3) in our case. Studies have also reported that the calcification is due to a circulatory disturbance in tumor tissue, or that tumor tissue tends to undergo hyaline degeneration and subsequent calcification when it contains a high percentage of collagen fibers [11].

“Nonepithelial tumor with calcification” is diverse [9], and the differential diagnosis includes conditions such as leiomyoma, angiofibroma, hemangioma, neurofibroma, schwannoma, and leiomyosarcoma [2]. In approaching such cases, the main difficulty is in defining whether the tumor is benign or malignant. In particular, because the treatment strategy and prognosis differ significantly between leiomyoma and leiomyosarcoma, their differentiation is very important. However, this is often difficult at the preoperative stage [2–4]. The presence of calcification and ossification is not considered helpful to rule out the diagnosis of malignancy [3, 12]. Moreover, cases of FDG-PET-positive uterine and esophageal leiomyomas have been reported [13, 14]. Preoperative FNAB needs to be performed more than once, although it was not effective in this case. Furthermore, depending on the tumor site, it is considered better to perform open biopsy or surgical resection carefully for definitive diagnosis of head and neck tumors with calcification and ossification that are difficult to differentiate, even using results of multiple examinations.

Proliferating spindle cells in a tangled arrangement are a characteristic histopathological feature of leiomyoma. In addition to histopathology, immunohistochemistry using *α*-SMA and h-caldesmon is also precise and reliable for definitive diagnosis [6]. *α*-SMA is most commonly used as a myogenic marker, and h-caldesmon is expressed exclusively in smooth muscle and is a highly specific marker for it [15]. Both are also helpful for differentiation from leiomyosarcoma [2]. In addition, it has been reported that the degree of mitotic activity can be one of the major distinguishing factors between leiomyoma and leiomyosarcoma [2, 9].

As in this case, leiomyoma can be diagnosed definitively and treated radically by surgical resection [1–4, 6, 8, 11, 12]. Generally, further treatment is unnecessary and its prognosis is excellent after surgery [1]. Regarding the mass in the upper mediastinum, we considered that it was most likely to be leiomyoma with calcification from the CT findings and his clinical course. Because its biopsy and resection are more invasive than those of submandibular leiomyoma, we accepted that they can be postponed until subjective symptoms such as dysphagia become remarkable, as for the submandibular leiomyoma.
